# Effects of Neurodevelopmental Therapy on Gross Motor Function in Children with Cerebral Palsy

**Published:** 2015

**Authors:** Sina LABAF, Alireza SHAMSODDINI, Mohammad Taghi HOLLISAZ, Vahid SOBHANI, Abolfazl Shakibaee

**Affiliations:** 1 Occupational therapist, Ebnesina Rehabilitation Clinic, Consulting Unit, Tehran, Iran.; 2 Department of Exercise Physiology, Exercise Physiology Research Center, Baqiyatallah University Medical Sciences, Tehran, Iran,; 3 Department of physical medicine and Rehabilitation, faculty of Medicine, Baqiyatallah University of Medical Sciences, Tehran, Iran.; 4 Exercise Physiology Research Center, Baqiyatallah University Medical Sciences, Tehran, Iran.

**Keywords:** Cerebral palsy, Children, Gross motor function, Neurodevelopmental treatment, Rehabilitation

## Abstract

**Objective:**

Neurodevelopmental treatments are an advanced therapeutic approach practiced by experienced occupational therapists for the rehabilitation of children with cerebral palsy. The primary challenge in children with cerebral palsy is gross motor dysfunction. We studied the effects of neurodevelopmental therapy on gross motor function in children with cerebral palsy.

**Materials & Methods:**

In a quasi-experimental design, 28 children with cerebral palsy were randomly divided into two groups. Neurodevelopmental therapy was given to a first group (n=15) with a mean age of 4.9 years; and a second group with a mean age 4.4 years (n=13) who were the control group. All children were evaluated with the Gross Motor Function Measure. Treatments were scheduled for three - one-hour sessions per week for 3 months.

**Results:**

We obtained statistically significant differences in the values between the baseline and post treatment in two groups. The groups were significantly different in laying and rolling (P=0.000), sitting (0.002), crawling and kneeling (0.004), and standing abilities (P=0.005). However, there were no significant differences in walking, running, and jumping abilities between the two groups (0.090).

**Conclusion:**

We concluded that the neurodevelopmental treatment improved gross motor function in children with cerebral palsy in four dimensions (laying and rolling, sitting, crawling and kneeling, and standing). However, walking, running, and jumping did not improve significantly.

## Introduction

In children with cerebral palsy (CP), lesions of the central nervous system (CNS) could cause motor-sensory impairments that progressively deteriorate over time (1). CP occurs in every 2/1000 and up to 2.5/1000 live births (2).The primary challenge for CP is gross motor dysfunction (GMD) (3, 4). In addition, the severity of limitation in gross motor function (GMF) among children with CP, the most common physical disability is highly variable (4, 5).The motor problems of CP arise fundamentally from CNS dysfunction, which interferes in the development of normal postural control against gravity and impedes normal motor development (5, 6, 7).Occupational therapy in children with CP is performed to avoid abnormal muscle tone and posture, to treat muscle and joint deformities, and to reduce motor and sensory disorders (8, 9).This approach for neurodevelopmental treatment for CP is the most widespread and is clinically accepted for targeting the CNS and the neuromuscular system. In view of the specific lesions in the CNS that ‘teaches’ the brain to improve motor performance skills and achieve ‘as near normal function as possible’ (10). The primary purpose of this approach is to correct abnormal postural tone and to facilitate more normal movement patterns for performing performance skills (11, 12).Although Neurodevelopment Therapy (NDT) is widely used by pediatric therapists in the treatment of children with CP, there is little research evidence regarding its efficacy in Iran. The purpose of this article is to present the basics of NDT for the rehabilitation of children with cerebral palsy.

## Materials & Methods

A total of 28 children with diplegic CP were selected from a population of individuals with CP who had been followed up at Ebnesina Rehabilitation Clinic. Our inclusion criteria were as follows: a diagnosis of cerebral palsy (patient’s diagnosis of CP confirmed by an expert pediatrician neurologist), no other severe abnormalities such as seizure, no participation in other therapeutic programs except for occupational therapy, between 2–6 years of age, and referred to the occupational therapy clinic of children with disabilities for a 3 month course of therapy. Our exclusion criteria were as follows: receipt of medical procedures likely to affect motor function such as botulinum toxin injections, orthopedic remedial surgery, mental retardation, or a learning disability. Participants were divided into a treatment group (15 subjects) and a control group (13 subjects).The GMF of patient’s were evaluated according to Gross Motor Function Measure (GMFM). The treatment group received and participated in neurodevelopment treatment for 3 months. In this study, the control group received home exercise, which included routine movement therapy (stretching, passive range of motion, and active range of motion) for 3 months by the parents and controlled by an occupational therapist. No statistically significant differences in physical and clinical characteristics of both groups were found before treatment (p>0.05).A convenience sample was used. Ethical approval was granted for the study and informed consent statements were signed by all the parents. One standardized validated measure of function was used as follows: the GMFM (GMFM-88) is a clinical measure designed to assesses gross motor abilities of children with CP in five dimensions: (1) Lie and Roll, (2) Sit, (3) Crawl and Kneel, (4) Stand, and (5) Walk, Run, and Jump (13). In children with CP, the GMFM has been shown to be sensitive to change during periods of therapy (9, 14, 15, 16).Assessments before and after treatment were completed by an occupational therapist. The treatment group that participated in the study underwent neurodevelopment treatments. In each session, exercises included patients sustaining themselves on their forearms and hands, sitting, crawling, semi-kneeling, and in standing positions supported by the occupational therapist until tone reduction was achieved. Balance and corrective reactions were developed by using a CP ball and tilt board after the children had acquired the skill of maintaining exercise positions. Ambulation training, appropriate to the motor development level (crawling, creeping, walking while in a semi-kneeling position, and walking between parallel bars) was given. Additionally, for this study, the NDT programme included passive stretching of the lower limb muscles (e.g. hamstrings, gastrocsoleus), followed by techniques of reducing spasticity and facilitating more normal patterns of movement while working on motor functions (10). The control group underwent the exercises (stretching, passive range of motion, and active range of motion) at home with their parents. The duration of the treatment for the two groups was three days a week for 3 months with each session being one hour. Statistical analysis: The normal distribution of variables was assessed with the Kolmogrov-Smirnov test. An independent sample t-test used for comparison of scores between the two groups. The pre-NDT and post-NDT intervention mean scores for each group were analyzed using a paired-sample t-test to determine whether there were any significant differences. Statistical analysis was performed with SPSS (version 17.0), with P-values less than 0.05 considered statistically significant.

## Results

A total of 28 children completed the entire duration of treatment for 3 months. A total of 15 subjects participated in the treatment group (7 girls, 8 boys; age range 2–6 years; mean age 4.9 years) and 13 subjects in the control group(7 girls, 6 boys; age range 2–5.5 years; mean age 4.4years). Table 1 provides the mean, standard deviation (SD), minimum and maximum scores for the GMFM-88, pre-treatment and post-treatment measures for both groups. The independent simple t-test showed significant improvements in GMFM scores between the two groups following neurodevelopment treatment in laying and rolling (P=0.000), sitting (P=0.002), crawling and kneeling (P=0.004), and standing abilities (P=0.005). However, there were no significant differences in walking, running, and jumping abilities between the two groups (0.090) (Table2). The paired t-test used for before and after measurements for the treatment group (NDT) revealed significant differences in GMFM-88 scores in laying and rolling, sitting, crawling, standing, and walking abilities (P> 0.05) (Table3). Nevertheless, in the control group, the paired-sample t-test revealed GMF significantly improved after intervention only in the rolling position (p<0.05). According to our results, as initially hypothesized, NDT intervention had a significantly positive effect on GMF in children with CP.

**Table 1:** Descriptive statistics in the treatment and control groups

**GMFM 88**				**Assessment**	**Group**
**Max**	**Min**	**SD**	**Mean**		
124	66	11.62	90.1	After treatment	**Treatment (NDT)**
132	105	10.41	112.6	Before treatment	
103	75	7.93	86.3	After treatment	**Control**
109	80	7.36	88.2	Before treatment

GMFM, Gross Motor Function Measure; Min, Minimum; Max, Maximum

**Table 2:** Gross Motor Function Measure in five positions–Independent simple t-test

**P**	**SD**	**Mean**	**Group**	**Ability**
0.000	13.8	23.2	Control	Lying and Rolling
	8.8	21	treatment	
0.002	8.5	34.3	Control	Sitting
	8.1	25.9	treatment	
0.004	5.2	20.7	Control	Crawling
	6.2	20	treatment	
0.005	4.5	19.7	Control	Standing
	9.2	17	treatment	
0.090	4	5	Control	Walking, running and jumping
4.4	4.3	treatment

**Table 3:** Pre and Post Gross Motor Function Measure in the treatment group (NDT)

**P**	**SD**	**GMFM Score**		**Ability**
		**Post**	**Pre**	
0.001	9.7	32.2	19.5	**Lying and Rolling**
0.04	4.9	36.7	32.2	**Sitting**
0.002	3.8	23.7	19.8	**Crawling**
0.010	1.2	19.7	17.8	**Standing**
0.04	1.5	6.3	4.9	**Walking** **, running and jumping**

## Discussion

Occupational therapy and neuro-rehabilitation approaches are important components in the treatment of children with CP (17). Improvement of GMF is one of the most important goals of treatment for children with CP. The primary aim of neurodevelopmental therapy is to promote GMF for children with CP. In this study, the focus was on neurodevelopmental therapy, which was administered for 3 months in children with spastic cerebral palsy (distribution of diplegia and quadriplegia), and their GMF as measured with the GMFM. The results of the present study showed significant improvement in rolling and laying, sitting, crawling, and standing abilities in children with spastic diplegia and quadriplegia after application of NDT using GMFM. The GMFM reflects the motor developmental sequence from birth to 5 years and necessarily includes activities that precede or that are prerequisites for the achievement of standing and walking. However, in the second group, which received no neurodevelopmental treatments, there was only significant improvement in rolling ability. Ketelaar et al showed significant differences in rolling, sitting, and kneeling after neurodevelopmental intervention (6). These results were consistent with the present study in that we show positive changes in laying and rolling, sitting, crawling, kneeling, and standing after NDT intervention. Shamsoddini also reported that use of the neurodevelopmental approach for the treatment of children with CP will lead to improvements in GMF (10). Fetters and Kluzik reported that the use of neurodevelopmental treatment for children with CP improved motor skills functionality (18). In this study, the data demonstrates in all of GMFs, that the NDT approach was statistically more significant than for the control group from the pre test to post test, because the NDT approach includes of improvements of GMF and postural stability. In addition, it may be that the treatment effects are most obvious in the postural set used for GMF and caused improved developmental ability (19). Akbari et al, a pre- and post-design study, showed that for GMF of subjects assessed with GMFM, the results indicated that a functional therapy program was effective in increasing GMF and improving daily activities in children with CP (20). Tsorlakis et al evaluated the effect of neurodevelopmental treatment on these functions in children CP. They used GMFM to measure the treatment effects and reported significant improvements for GMF from the GMFM total scores (21). Also, Arndt et al evaluated the efficacy of a neurodevelopmental treatment based sequenced trunk activation protocol for changes in the GMF of infants with posture and movement dysfunction, their results showed that the NDT-based protocol group made significantly more progress than the control group from pretest to posttest (22). To mention the limitations of study, although GMFM provides a great deal of data, it is complex and time consuming for staff to gather and analyze. Another limitation has been the interpretation of the GMFM total score. In conclusion, our findings show that neurodevelopmental treatments may be effective in improving in GMF after intervention in children with CP. There were a number of issues arising from this study, which suggest directions for future investigations. However, it is clear that further studies are needed to support the results of this study through replication and to investigate other issues related to the effectiveness of the treatment.
